# False-Positive HIV Serology in APECED: A Potential Diagnostic Pitfall

**DOI:** 10.1007/s10875-026-02064-4

**Published:** 2026-08-18

**Authors:** Joseph Pechacek, Taura Webb, Michail S. Lionakis

**Affiliations:** Fungal Pathogenesis Section, LCIM, NIAID, NIH, Bethesda, MD USA

To the Editor:

Autoimmune PolyEndocrinopathy-Candidiasis-Ectodermal Dystrophy (APECED), also known as Autoimmune polyendocrine syndrome type-1, is a monogenic syndrome caused by deleterious variants in the Autoimmune Regulator (*AIRE*) gene that impair thymic negative selection and lead to multiorgan autoimmunity [1]. The disease is characterized by chronic mucocutaneous candidiasis (CMC) and multiorgan autoimmunity that are driven by excessive interferon-γ (IFN-γ) responses and is notable for the presence of a broad and diverse autoantibody repertoire, including high-titer autoantibodies against type-I interferons and type-17 cytokines [1].

Modern fourth-generation Human Immunodeficiency Virus (HIV) screening assays, which detect antibodies to HIV-1/2 and the p24 HIV antigen, have high sensitivity and specificity [2]. Reactive screening tests are followed by HIV-1/2 antibody differentiation assays, with nucleic acid amplification testing performed when results are discordant. Although these assays have excellent performance characteristics, false-positive results may occur, particularly in settings associated with immune activation or autoantibody production.

False-positive HIV serologic testing has been described in several polygenic autoimmune conditions, including systemic lupus erythematosus [3] and in certain infection settings such as recent SARS-CoV-2 infection, likely due to cross-reactive antibodies. Reports in inborn errors of immunity are exceedingly rare. To our knowledge, the only prior report of false-positive HIV serologic testing involved a patient with chronic granulomatous disease, who presented with inflammatory colitis and hypergammaglobulinemia, in whom polyclonal antibody responses were proposed to account for assay interference [4].

We describe an 18-year-old Italian woman with genetically-confirmed APECED (homozygous *AIRE* variant c.415 C > T) with manifestations including CMC, hypoparathyroidism, adrenal insufficiency, autoimmune hepatitis, enamel hypoplasia, asplenia, keratoconjunctivitis, and hypogonadism. She was enrolled in a National Institutes of Health (NIH) prospective natural history study (NCT01386437) and underwent routine clinical and laboratory evaluations including HIV screening.

A fourth-generation HIV antigen/antibody screening assay returned reactive. The patient reported no fevers, myalgias, or other symptoms suggestive of acute retroviral infection and had no high risk epidemiologic factors for HIV. Physical examination revealed no lymphadenopathy or rash. Laboratory evaluation demonstrated a IgG level in the high-normal range (1429 mg/dL; normal, 658–1534 mg/dL), elevated IgA (520 mg/dL; normal, 53–287 mg/dL), normal IgM and IgE levels, negative rheumatoid factor, and negative pregnancy testing.

Confirmatory testing was subsequently performed. HIV1/2 antibody differentiation assays were negative for both HIV-1 and HIV-2 antibodies, and nucleic acid testing for HIV RNA was undetectable for both HIV-1 and HIV-2, most likely consistent with a false-positive initial screening result. As there is a small population of HIV infected patients deemed elite controllers who may similarly present with a positive HIV screen and negative HIV RNA without antiretroviral therapy [5], peripheral blood mononuclear cells (PBMCs) were isolated, tested for HIV DNA, and found to be negative for the HIV genes *env* and *gag* and for the long terminal repeat region (LTR), confirming that the initial screening result represented a false-positive HIV test. Consistent with the absence of HIV infection, the patient had a preserved CD4^+^ T cell count (1404 cells/µL; 54% of lymphocytes).

Although the precise mechanism underlying the false-positive HIV screening test in this patient remains uncertain, patients with APECED develop an unusually broad array of autoantibodies, including antibodies targeting cytokines and diverse tissue antigens, with sera reported to recognize a large number of self-antigens. It is therefore plausible that non-specific antibody binding or cross-reactivity with HIV antigens used in screening immunoassays may account for the observed result. Similar mechanisms have been proposed in other autoimmune conditions associated with false-positive HIV testing [3, 4].

This observation suggests that individuals with monogenic immune dysregulation syndromes such as APECED may be at risk of false-positive HIV screening results though further study is needed to establish the rate of false-positive testing in this population. Recognition of this potential laboratory artifact is important, as reactive HIV screening tests may cause significant patient anxiety and prompt extensive diagnostic evaluations. Clinicians caring for patients with APECED and other inborn errors of immunity characterized by immune dysregulation should therefore interpret reactive screening assays cautiously and rely on confirmatory antibody differentiation and nucleic acid testing to establish or exclude HIV infection.

## Data Availability

No datasets were generated or analysed during the current study.
